# A Bibliometric and Knowledge‐Map Analysis of Macrophage Polarization in Insulin Resistance From 1999 to 2023

**DOI:** 10.1002/iid3.70048

**Published:** 2024-10-28

**Authors:** Chuning Lin, Yuan Chen, Yao Ge, Huimin Niu, Xinyi Zhang, Feng Jiang, Chuyan Wu

**Affiliations:** ^1^ Department of Rehabilitation Medicine The First Affiliated Hospital of Nanjing Medical University Nanjing Jiangsu Province China; ^2^ Department of Neonatology Obstetrics and Gynecology Hospital of Fudan University Shanghai Jiangsu Province China

**Keywords:** bibliometric analysis, CiteSpace, insulin resistance, macrophage polarize, research hotspots

## Abstract

**Background:**

Despite numerous studies confirming the association between insulin resistance (IR) and macrophage polarization, there is a lack of bibliometric analysis in this area. Therefore, our objective is to conduct a comprehensive analysis of published literature and identify potential future research trends using bibliometrics.

**Method:**

Publications on the topic of macrophage polarization in IR were gathered from the Web of Science Core Collection database (WoSCC) spanning the years 1999–2023. Bibliometric analysis and visualization were conducted using VOSviewers, CiteSpace, the R package “bibliometrix” and Tableau Public.

**Result:**

A total of 3435 articles published between 1999 and 2023 were included in the analysis. These articles originated from 75 countries, with the United States and China leading in contributions. The top five research institutions are the University of California, San Diego, Harvard University, the University of Michigan, Shanghai Jiao Tong University, and Huazhong University of Science and Technology. In this research domain, *Diabetes* is the most frequently published journal, and the *Journal of Clinical Investigation* is the most co‐cited. Among the 19,398 authors contributing to these publications, Lumeng CN. not only authored the most papers but also received the highest number of co‐citations. “Insulin resistance” emerges as a primary keyword in the analysis of emerging research hotspots.

**Conclusion:**

For the first time, bibliometric methods have been employed to conduct a comprehensive summary of papers relevant to macrophage polarization in IR. This study aims to identify the current research direction and future research hotspots, offering valuable guidance and insights for scholars in the field.

## Graphical abstract

We conducted an all‐sided analysis of the published articles on macrophage polarization in IR spanning from 1999 to 2023, aiming to understand the current research progress, and trends, and anticipate future developments in the field.

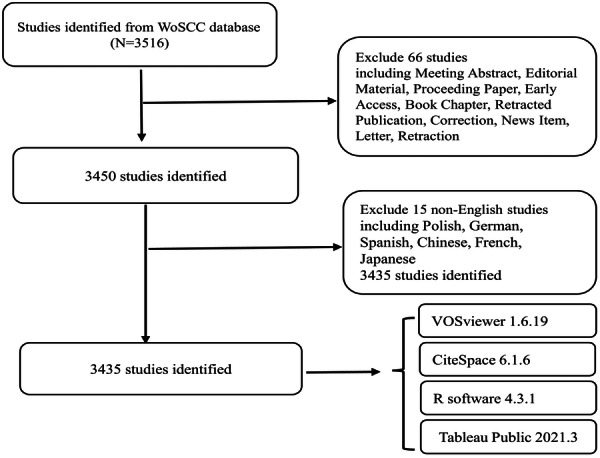

## 1 Introduction

Insulin resistance is physiologically defined as a state in which the responsiveness of insulin‐target tissues to high physiological insulin levels is reduced. It is considered a pathogenic factor in many modern diseases, including metabolic syndrome, nonalcoholic fatty liver disease (NAFLD), atherosclerosis, and type 2 diabetes [1, 2, 3]. Insulin resistance precedes nonphysiological elevations in blood glucose levels and is a key pathogenic factor in many metabolic diseases [4, 5, 6]. Chronic low‐grade inflammation and activation of the immune system are involved in the pathogenesis of insulin resistance. Adipose tissue, liver, muscle, and pancreas are themselves sites of inflammation. Infiltration of macrophages and other immune cells is observed in these tissues, with the cell populations shifting from anti‐inflammatory to proinflammatory. These cells are crucial for the production of proinflammatory cytokines, which interfere with insulin signaling in peripheral tissues through autocrine and paracrine mechanisms or induce β‐cell dysfunction and subsequent insulin deficiency. Research shows that IR is closely related to macrophages [7], which are thought to be an important source of inflammatory cytokines [8]. When macrophage polarization occurs, mature macrophages develop distinct functional phenotypes in response to specific microenvironmental stimuli [9, 10, 11]. This process can trigger inflammatory and immune responses [12, 13], potentially exacerbating insulin resistance [14, 15, 16].

According to the functional state of macrophages after polarization, macrophage polarization can be categorized into M1 and M2 types [17]. M1 macrophages, which are classically activated, are proinflammatory and can be polarized by lipopolysaccharide (LPS) alone or in combination with Th1 cytokines (e.g., IFN‐γ, GM‐CSF), producing proinflammatory cytokines [18]. M2 macrophages which are alternatively activated have anti‐inflammatory and immunoregulatory effects are polarized by Th2 cytokines, and produce anti‐inflammatory cytokines [19, 20, 21]. With the gradual deepening of the study of macrophages, the M1/M2 classification is considered an oversimplified approach that does not adequately cover the total spectrum of macrophage phenotypes [22, 23]. Alternatively, there is an emerging spectrum of activation states such as M2a associated with the initial phase of tissue repair [24], M2b described as a “middle‐of‐the‐road,” M2c characterized by high expression of anti‐inflammatory cytokines and immune suppression, among other subsets [25, 26]. Most current studies of the emerging spectrum of polarization have used mice [27, 28, 29]. However, there are still differences in macrophage polarization between mice and humans, including variations in protein expression, metabolic pathways, immune response functions, responses to stimuli, and adaptation to microenvironments [30, 31]. These differences highlight the necessity of taking species‐specific variations into account when applying findings from preclinical animal models to human disease contexts.

Bibliometrics is an interdisciplinary statistical method [32] that enables researchers to analyze publications within a specific field and process data through mathematical means [33, 34, 35], considering both quantitative and qualitative variables [36, 37]. To date, there have been bibliometric studies on the trends of macrophage polarization in diabetes, rheumatoid arthritis, atherosclerosis, COPD, and tumors. However, the exploration of macrophage polarization in IR has not been undertaken before, presenting significant potential. To advance knowledge in this area, we performed a bibliometric analysis of macrophage polarization in IR from 1999 to 2023, intending to understand current research progress and trends and predict future developments in the field.

## 2 Methods

### 2.1 Search Strategies

**Figure 1:**
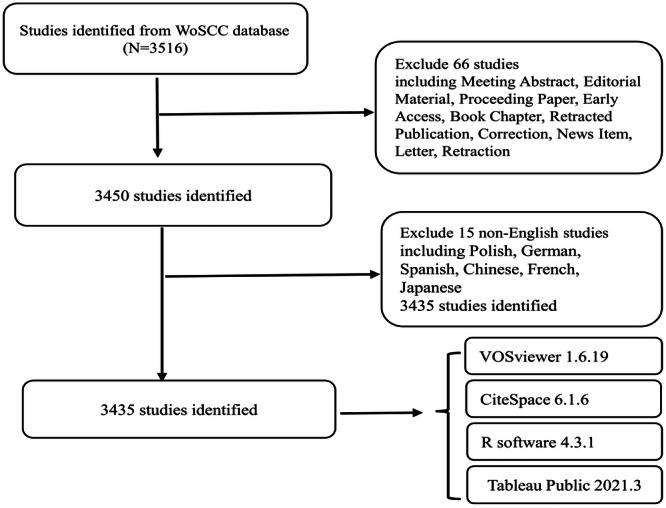
Flowchart of the screening process.

The literature retrieved for our analysis was sourced from the Web of Science Core Collection database (WoSCC) [38], recognized as one of the most commonly used platforms for academic and bibliometric studies. The search criteria employed to identify articles were as follows: TS = (macrophage polarization) OR (Activation, Macrophage) OR (Activations, Macrophage) OR (Macrophage Activations) AND TS = (Insulin Resistance) OR (Resistance, Insulin) OR (Insulin Sensitivity) OR (Sensitivity, Insulin). A total of 3516 studies were identified regarding macrophage polarization in IR, 66 of which were excluded due to their document type issues. The publication type was restricted to “Article” and “Review.” Of the remaining 3450, 15 were excluded because they were non‐English studies. The search process is presented in a flowchart diagram. (Figure 1).

### 2.2 Data Analysis

VOSviewer (Version 1.6.19) is a tool designed for the analysis and visualization of bibliometric networks [39, 40, 41], encompassing journals, researchers, or individual publications, and considering various relationships [42, 43]. In this study, we primarily utilized this software for the following analyses: authors and co‐cited authors, sources (journals) and co‐cited sources (journals), co‐occurrence of all keywords, analysis of countries, and analysis of organizations (institutions).

CiteSpace (Version 6.1.6) is a visual analytics tool created by Professor Chaomei Chen for the analysis of trends and patterns in scholarly literature within a specific research field [44]. In this study, the software was employed to generate JCR journal maps and analyze references with the strongest citation burst.

The R package “bibliometrix” (Version 4.3.1) [45] serves as a tool for quantitative research in scientometrics and bibliometrics. It offers a variety of routines for importing bibliographic data, such as from the WoSCC, conducting bibliometric analysis, and constructing networks [46] for co‐citation, coupling, scientific collaboration, and co‐word analysis.

Tableau Public (Version 2021.3) [47] is a free platform featuring millions of captivating online data visualizations for exploration, creation, public sharing, and learning [48].

## 3 Results

### 3.1 The Trend of Publication Outputs

**Figure 2:**
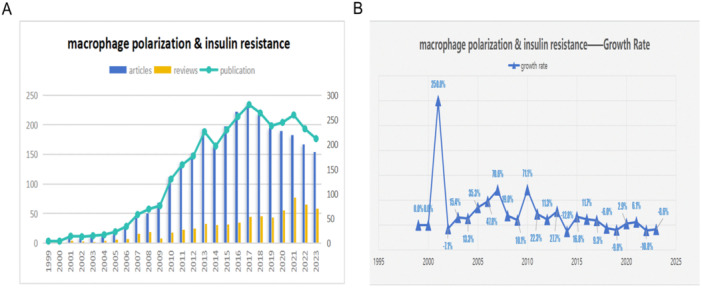
(A) Comparison of articles and reviews of publishing. Blue bars represent the number of articles published each year, while yellow bars indicate the number of reviews. (B) The growth rate of macrophage polarization in IR. Each triangle on the line graph denotes a year, with the adjacent labels indicating the annual growth rate of publications.

Utilizing search strategies, a total of 3435 studies on macrophage polarization in IR were identified from 1999 to 2023, comprising 2777 articles and 658 reviews. To show the comparison between the articles and reviews more intuitively, we drew the bar graph based on the quantities of both. The number of articles is much greater than that of reviews, except in 2000 (Figure 2A). The overall output demonstrated an upward trajectory, peaking in 2017 with 281 publications. Subsequently, there was fluctuation from 2017 to 2022, followed by a slight decline to 212 in 2023. Considering the impact of the COVID‐19 pandemic, this decrease was both anticipated and reasonable. To further analyze these trends, we examined the growth rate, which measures the increase in numbers compared to the total from the previous year. The growth rate soared in 2001 and then sharply declined in 2002, after which it exhibited some fluctuations (Figure 2B).

### 3.2 Analysis of Countries and Regions

A total of 75 countries/regions have contributed publications on macrophage polarization in IR. The top 10 countries are primarily located in North America, Asia, and Europe. We can observe that among the top 10 countries with the highest number of article publications, Germany (*N* = 164, 71.62%) has the highest proportion of collaborative papers in this field with other countries, closely followed by France (*N* = 101, 65.58%) (Table 1). It is evident when analyzing Table 2 alone that the USA is a powerful initiator of international collaboration that collaborates most with China.

**Table 1:** Top 10 countries and their collaboration in search of macrophage polarization in IR.

Rank	Country	Document	Percentage	Collaboration frequency	Collaboration percentage
1	USA	1237	37%	710	57.40%
2	China	729	22%	136	18.66%
3	Japan	336	10%	59	17.56%
4	Germany	229	7%	164	71.62%
5	South Korea	220	7%	17	7.73%
6	France	154	5%	101	65.58%
7	Canada	149	4%	36	24.16%
8	UK	138	4%	81	58.70%
9	Spain	122	4%	62	50.82%
10	Italy	108	3%	49	45.37%

**Table 2:** Top 10 collaboration relationships in search of macrophage polarization in IR.

Rank	Collaboration from	Collaboration to	Frequency
1	USA	China	181
2	USA	Germany	58
3	USA	Korea	55
4	USA	Japan	48
5	USA	UK	47
6	USA	Canada	37
7	USA	France	27
8	USA	Spain	25
9	France	UK	24
10	USA	Netherlands	24

**Figure 3:**
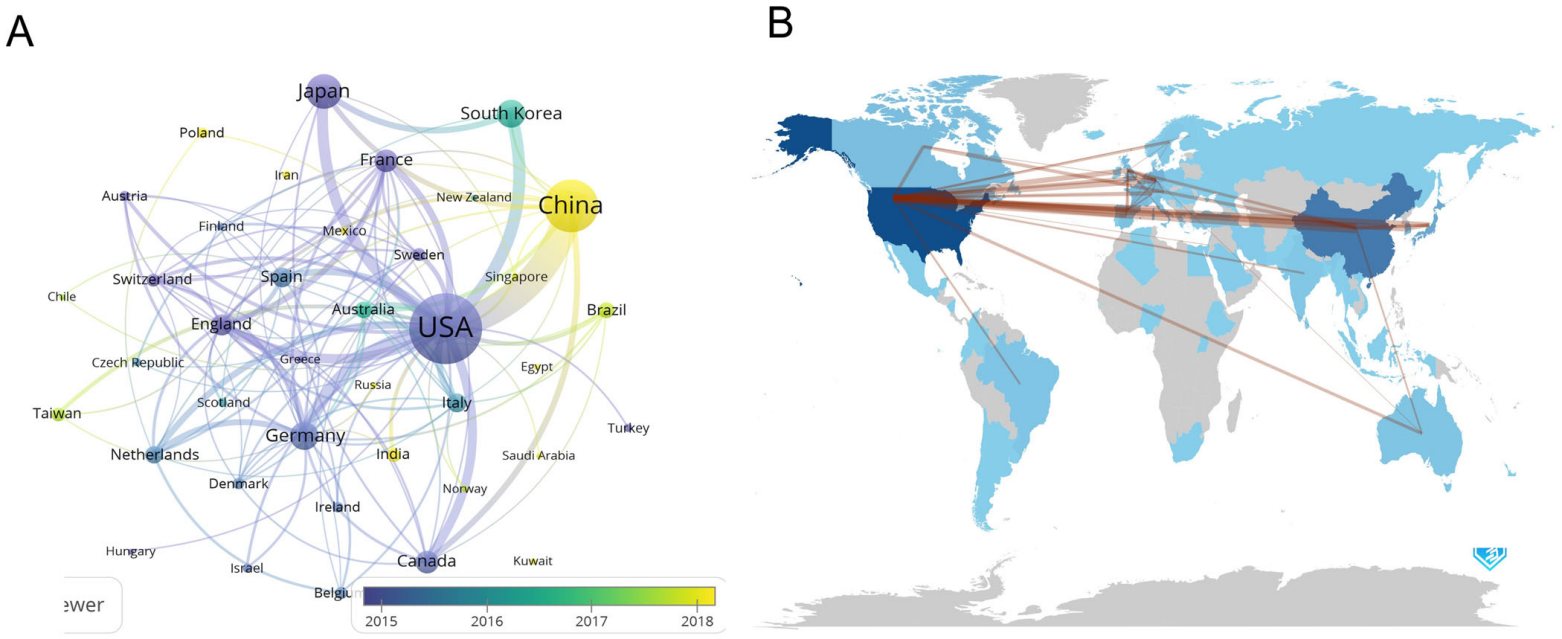
(A) The visualization of the number of countries' publications. Node size reflects publication count, color represents temporal progression, and line thickness indicates the strength of collaboration between countries. (B) Collaboration world map. Red lines connecting countries signify cross‐border collaborations in macrophage polarization research related to insulin resistance.

Subsequently, we utilized VOSviewer and Tableau Public to filter and visualize 39 countries, applying a criterion that each country must have at least 10 documents (Figure 3A). At the center of this network, the United States emerges as a prominent node, signifying its significant contribution to the field through a high number of publications. Other notable nodes include the People's Republic of China, South Korea, and Japan, which are highly interconnected, indicating robust collaborations and interactions in macrophage polarization research. The network also reveals clusters of countries that are closely linked, such as the grouping comprising Australia, Brazil, and India, which are positioned on the periphery of the main network.

To further show the collaboration and relationships in the publication outputs of each country, we visualized a collaborative network through a world map and a table that shows the raw data directly. The world map depicts various countries with their respective locations on a world map which are labeled in deep blue, with their national boundaries outlined in a lighter shade of blue, providing a clear visual representation of their geographical locations. The network of lines connecting these countries represents the cross‐border collaborations in this field of research, indicating the existence of collaborative efforts between researchers from different regions, highlighting the interconnectedness and global nature of this research (Figure 3B).

### 3.3 Analysis of Institution

**Figure 4:**
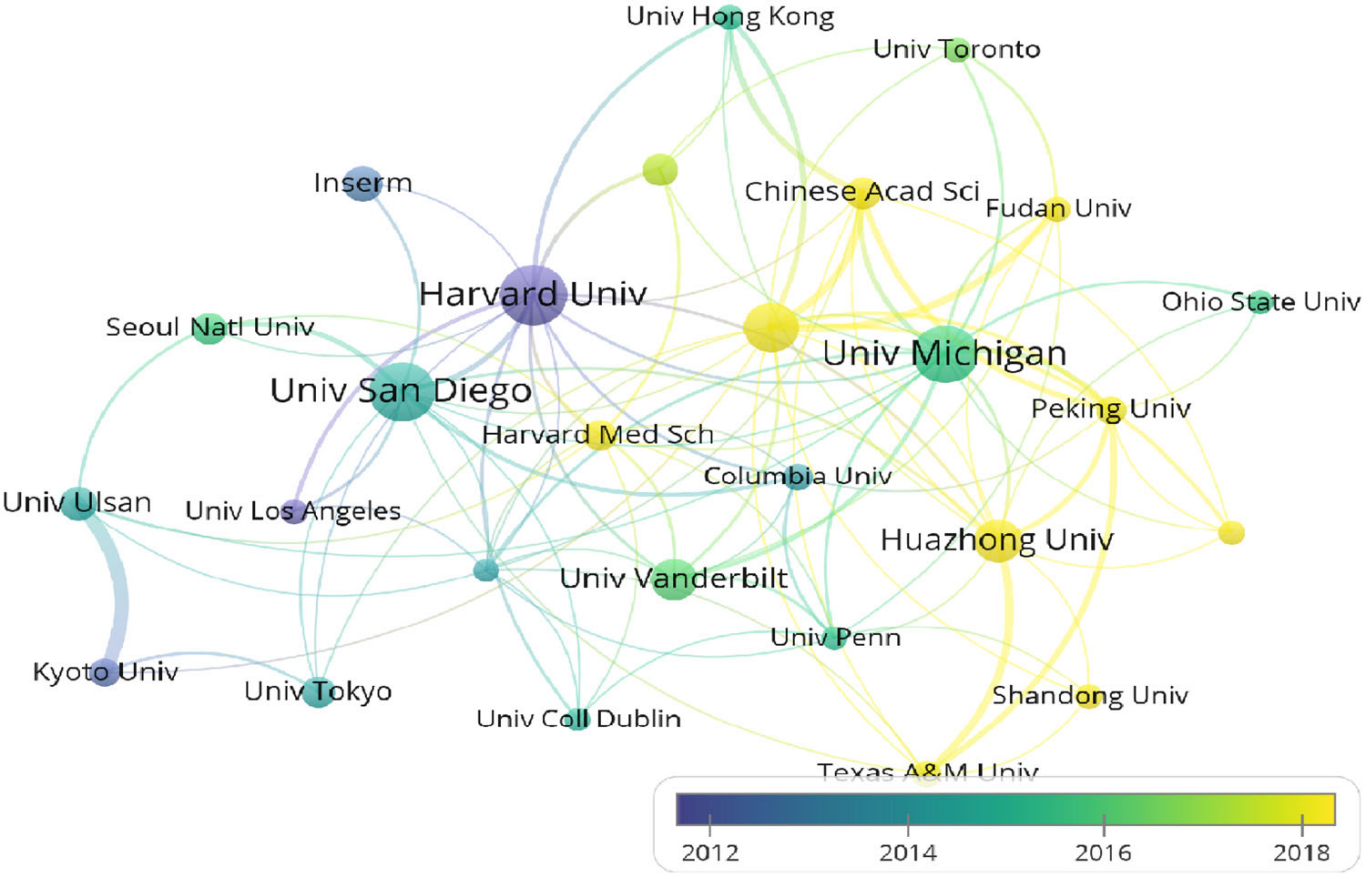
The visualization of institutions on the research of macrophage polarization in IR. Nodes represent institutions, with size indicating publication frequency. The color gradient reflects the year of the first publication.

A total of 3178 institutions participated in research on macrophage polarization in IR. We employed VOSviewer to generate a visualization map (Figure 4). A prominent feature of this network is the centrality of Harvard University, represented by the largest node. This positioning highlights its significant contribution and extensive collaboration with other institutions in the field. Other notable nodes include the University of California, San Diego, and the University of Michigan, indicating their substantial involvement in the research. The dense connections between these institutions suggest a robust collaborative effort in the area of macrophage polarization in insulin resistance.

Subsequently, we compiled a list of the top 10 institutions with the highest publication output, with Seoul National University and Sao Paulo University tied for 10th place (Table 3). Notably, the top 10 institutions are based in the USA (1st, 2nd, 3rd, 6th), China (4th, 5th, 9th), South Korea (8th, 10th), France (7th), and Brazil (10th). Harvard University stood out as the leading institution in terms of productivity in the field, closely followed by two other institutions in the USA.

**Table 3:** Top 10 institutions' names, document quantity, and countries.

Rank	Institution	Documents	Countries
1	Harvard Univ	72	USA
2	Univ Calif San Diego	70	USA
3	Univ Michigan	69	USA
4	Shanghai Jiao Tong Univ	58	China
5	Huazhong Univ Sci & Technol	51	China
6	Vanderbilt Univ	50	USA
7	Inserm	42	France
8	Univ Ulsan	40	South Korea
9	Chinese Acad Sci	39	China
10	Seoul Natl Univ	38	South Korea
10	Sao Paulo Univ	38	Brazil

### 3.4 Analysis of Journals and Co‐Cited Journals

**Figure 5:**
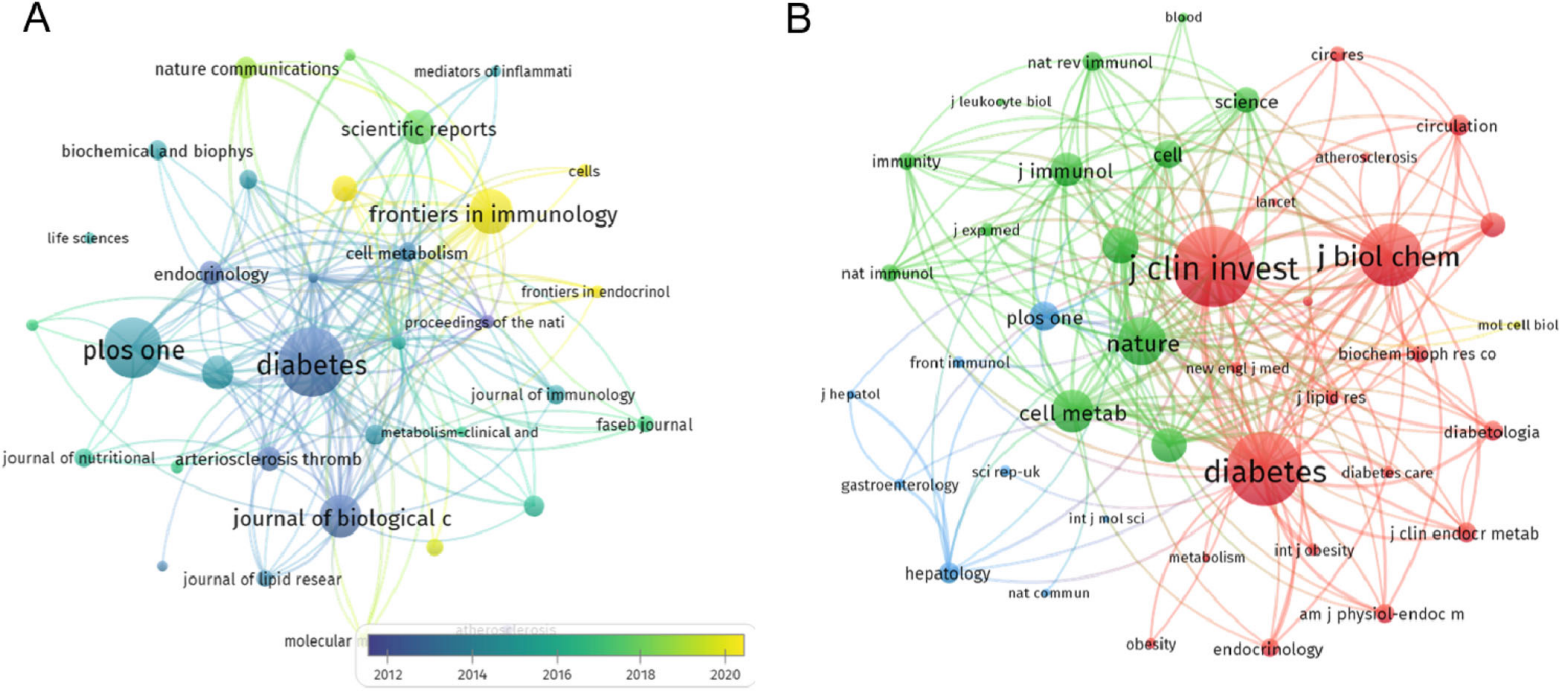
(A) The visualization of journals. Nodes symbolize individual journals, with lines indicating citations or collaborative relationships between them. The color gradient (blue to yellow) signifies increasing years. (B) The co‐cited journals. The size and color of the nodes highlight the number of co‐citations, while the lines represent the co‐citation relationships between them.

The research focused on macrophage polarization in IR is distributed across 749 journals. Using VOSviewer, we generated a visualization map for the journals, filtering and displaying 150 journals that have published at least 7 relevant studies (Figure 5A). Notably, *Diabetes* published the highest number of papers (*N* = 131, 3.91%), followed by *PLoS ONE* (*N* = 116, 3.46%), *Frontiers in Immunology* (*N* = 85, 2.54%), and *Journal of Biological Chemistry* (*N* = 82, 2.45%) (Table 4).

**Table 4:** Top 10 journals and co‐cited journals.

Rank	Journal	Document	Percentage	Rank	Journal	Co‐cited
1	Diabetes	131	3.91%	1	Journal of Clinical Investigation	9710
2	Plos One	116	3.46%	2	Diabetes	8861
3	Frontiers In Immunology	85	2.54%	3	Journal of Biological Chemistry	7475
4	Journal of Biological Chemistry	82	2.45%	4	Nature	5797
5	Scientific Reports	65	1.94%	5	Cell Metabolism	5077
6	American Journal of Physiology‐Endocrinology and Metabolism	64	1.91%	6	Proceedings of the National Academy of Sciences of the United States of America	4414
7	International Journal of Molecular Sciences	51	1.52%	7	Nature Medicine	4217
8	Arteriosclerosis Thrombosis and Vascular Biology	47	1.40%	8	Journal of Immunology	3962
9	Endocrinology	45	1.34%	9	Plos One	3570
10	Nature Communications	42	1.25%	10	Cell	3380

Next, we used VOSviewer to create a visualization map of co‐cited journals. Among the 6586 co‐cited journals, we required each journal to be cited at least 350 times, which filtered out 92 journals (Figure 5B). As shown in Table 4, the top 10 co‐cited journals each had over 3000 citations. The most co‐cited journal, *Journal of Clinical Investigation*, was cited 9710 times, followed by *Diabetes* (total citations = 8861) and *Journal of Biological Chemistry* (total citations = 7475) (Table 4).

**Figure 6:**
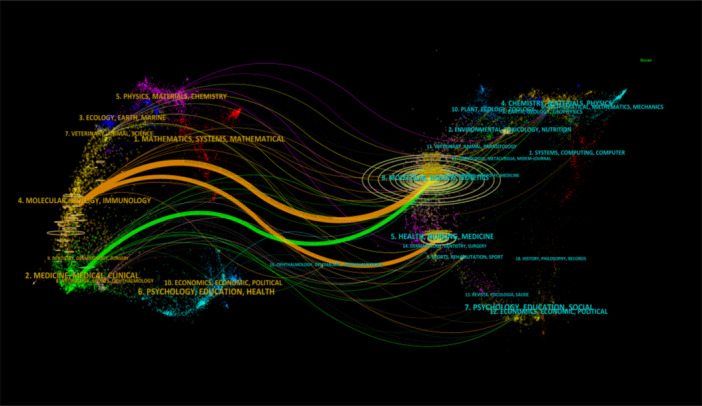
The dual‐overlay journal map on the research of macrophage polarization in IR.

To further analyze the relationships between journals and their cited journals, we used CiteSpace to create a dual‐map overlay of journals. This map not only distinguishes between citing journals (on the left) and cited journals (on the right) but also visualizes two main citation paths—the orange curve and the green curve (Figure 6).

### 3.5 Analysis of Authors and Co‐Cited Authors

Since 1999, there have been 19,389 researchers actively engaged in research related to IR and macrophage polarization who have published their findings. Using data from the WoSCC, we identified the top 10 authors in this field (Table 5). Lumeng. CN from the University of Michigan ranked highest, having published 27 papers, including 24 articles and 3 reviews, followed by Rina.Yu (*N* = 24) and Jerrold M. Olefsky (*N* = 21).

**Table 5:** Top 10 authors and their institution.

Rank	Author	Article	Review	Document	Institution
1	Lumeng, CN	24	3	27	University of Michigan
2	Yu, R	25	0	25	University of Ulsan
3	Olefsky, JM	20	2	22	University of California San Diego
4	Kawada, T	21	0	21	Kyoto University
4	Roche, HM	19	2	21	Roche, Helen M.
6	Ota, T	15	4	19	Kanazawa University
7	Hasty, AH	14	4	18	Vanderbilt University
7	Rajagopalan, S	17	1	18	University Hospitals of Cleveland
9	Nagashimada, M	14	3	17	Kanazawa University
9	Xu, AM	16	1	17	University of Hong Kong

**Figure 7:**
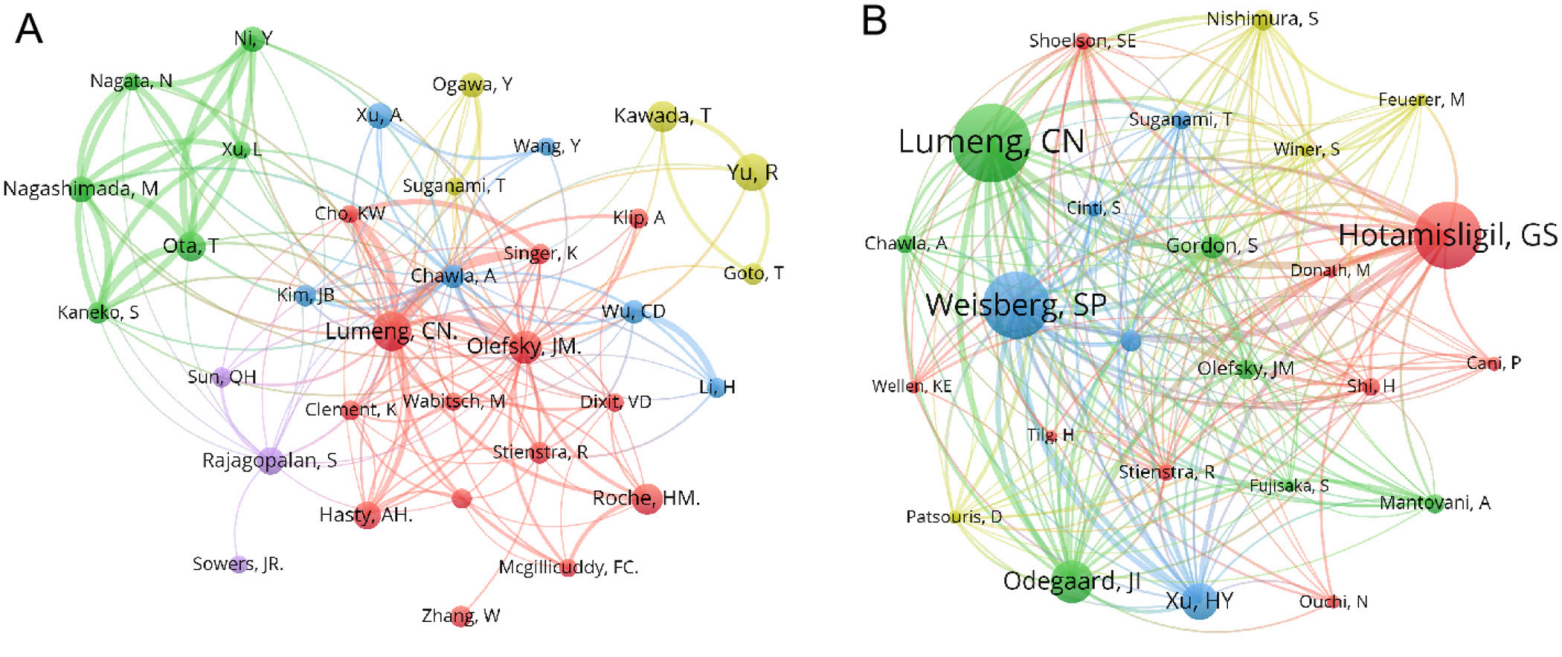
(A) The visualization of authors and (B) co‐cited authors.

To enhance visualization, we used VOSviewer to create an author visualization map, which includes 352 authors, with the filter criterion being at least five related publications (Figure 7A). Lumeng CN has the largest node, indicating the highest number of published articles, and Jerrold M. Olefsky shows significant connections with surrounding authors. Additionally, we analyzed co‐cited authors. Among the 71,702 co‐cited authors, Lumeng CN, who has the highest publication count, also received the most co‐citations (*N* = 1536). Besides Lumeng CN, two other authors, Weisberg SP (*N* = 1312) and Hotamisligil GS (*N* = 1311), had over 1000 co‐citations (Table 5). Furthermore, we generated a visualization map of co‐cited authors using VOSviewer, displaying all authors with at least 80 co‐citations (Table 6 and Figure 7B).

**Table 6:** Top 10 co‐cited authors.

Rank	Author	Co‐cited
1	Lumeng, CN	1536
2	Weisberg, SP	1312
3	Hotamisligil, GS	1311
4	Odegaard, JI	834
5	Xu, HY	717
6	Gordon, S	481
7	Nishimura, S	417
8	Kanda, H	414
9	Olefsky, JM	401
10	Mantovani, A	385

### 3.6 Analysis of Co‐Cited Reference and Citation Bursts

**Figure 8:**
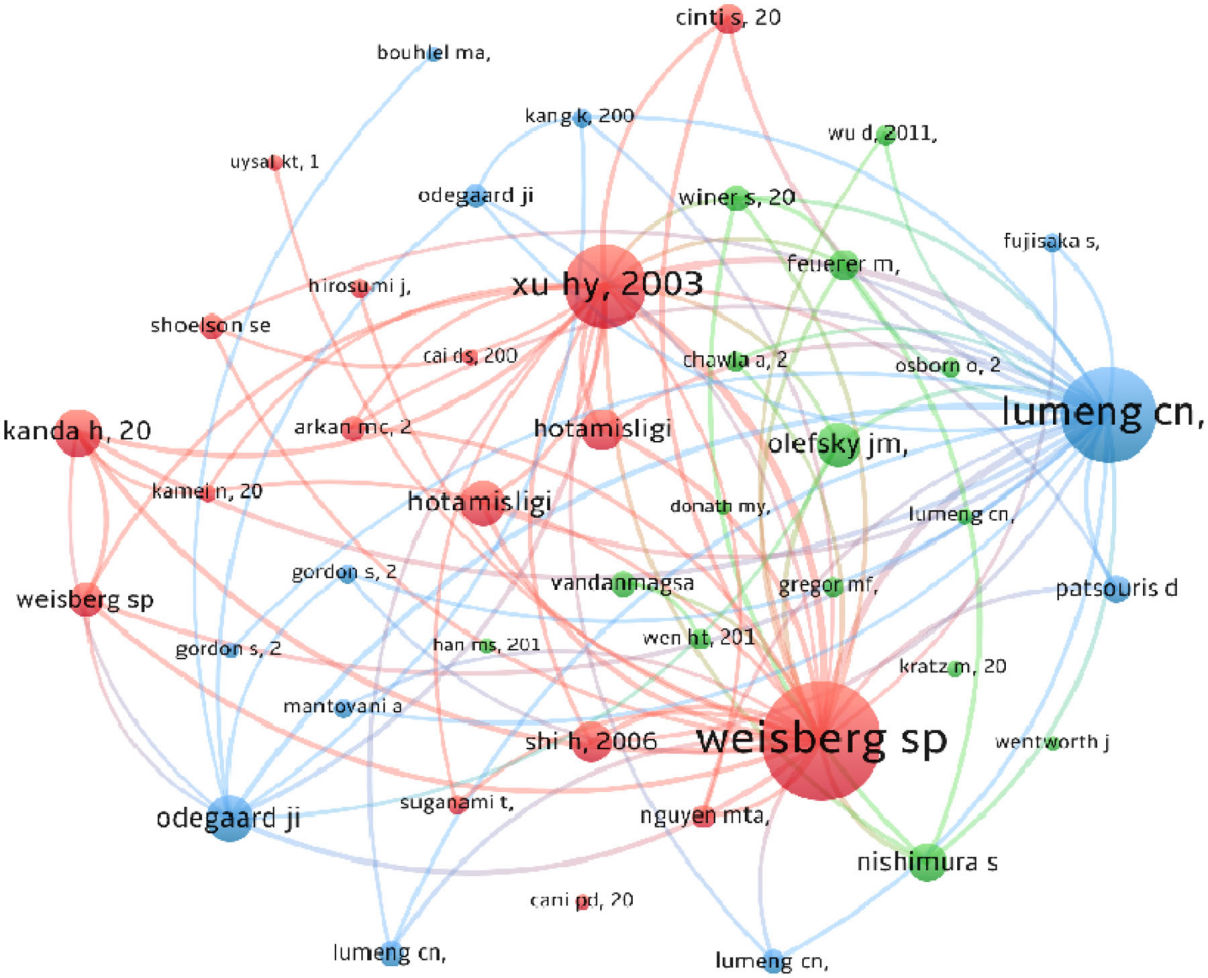
The visualization of co‐cited references. Nodes represent citations, with size indicating frequency. Line thickness signifies the strength of co‐citation relationships, revealing their connections.

From 1999 to 2023, there have been 114,118 references related to research on insulin resistance and macrophage polarization. To enhance clarity, we selected references with over 90 co‐citations and used VOSviewer to create a visualization map (Figure 8). The map shows that the most frequently co‐cited reference in this field is “Weisberg SP, 2003, Journal of Clinical Investigation” (*N* = 1010), followed by “Lumeng CN, 2007, Journal of Clinical Investigation” (*N* = 813) and “Xu HY, 2003, Journal of Clinical Investigation” (*N* = 702).

**Figure 9:**
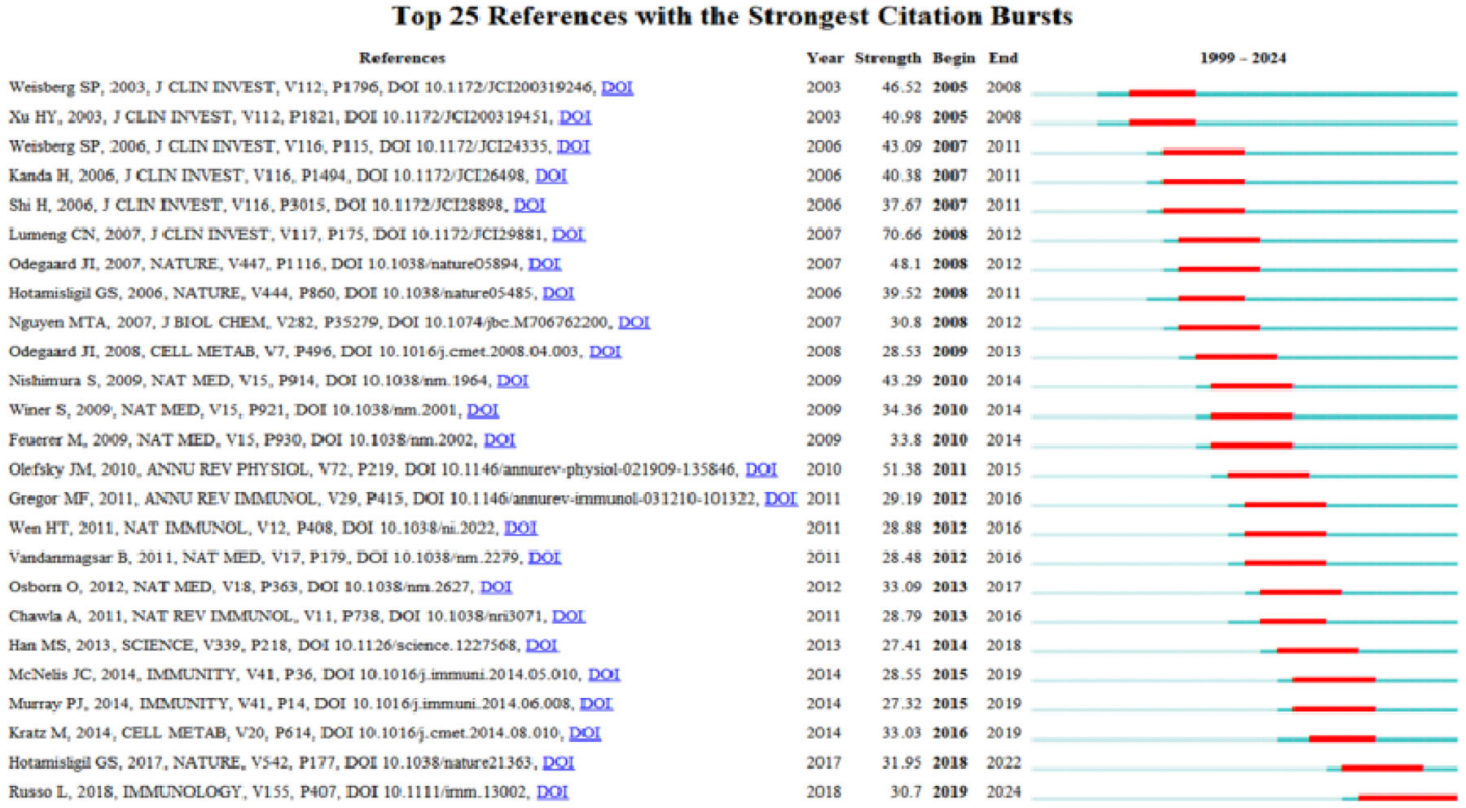
Top 25 references with the strongest citation bursts. The blue line represents the time from its first appearance before 2024, and the red line represents the burst time.

To depict which references were frequently cited over specific periods, we utilized CiteSpace to generate a visual representation highlighting the top 25 references with significant citation bursts. Citation bursts denote sudden spikes in citations during particular periods, indicating notable growth in reference influence, with years of substantial citations marked in red (Figure 9). This analysis spans citation bursts from 2005 to 2023 and underscores the enduring popularity of macrophage polarization in IR as a research topic. The research showing the strongest citation burst is titled “Obesity is associated with macrophage accumulation in adipose tissue,” authored by Weisberg SP in December 2003, with citation bursts observed from 2003 to 2008 (strength = 46.52). Overall, citation burst strengths for the top 25 references ranged from 46.52 to 30.7, and these bursts persisted for durations of 3 to 5 years.

### 3.7 Analysis of Keywords and Hot‐Spot

By analyzing high‐frequency keywords, we can directly identify hotspots in a specific field. Consequently, we opted to use VOSviewer for visualizing keywords. However, some keywords may have different spellings and syntactic orders in different countries, necessitating the combination of certain keywords. For instance, the terms “Activation, Macrophage,” “Activations, Macrophage,” and “Macrophage Activations” were consolidated into “macrophage polarization.” Similarly, the terms “Resistance, Insulin,” “Insulin Sensitivity,” “Resistance, Insulin,” “Sensitivity, Insulin,” “Insulin‐resistance,” and “Insulin resistance” were amalgamated into “Insulin Resistance.” The terms “macrophages,” “Macrophage,” and “Macrophages” were unified into “macrophage.”

**Figure 10:**
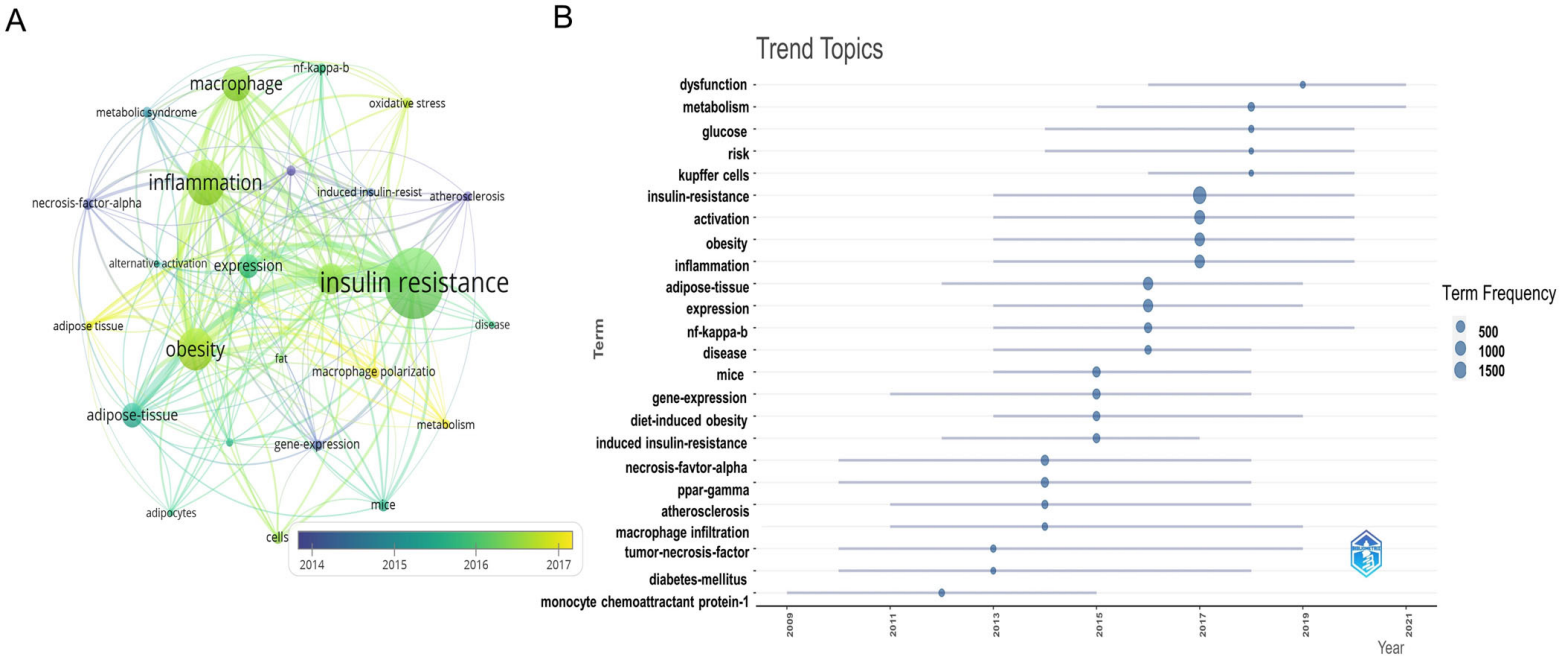
(A) The visualization of keywords. Nodes represent keywords, with size indicating frequency of occurrence. Interconnecting lines show relationships between terms. (B) The trend topics in search of macrophage polarization in IR. The x‐axis represents the years, while the y‐axis indicates various trend topics. The size of the circles corresponds to the frequency of occurrence of each topic.

Based on these adjustments, we compiled a table of the top 10 keywords (Table 7) and generated an analysis chart using VOSviewer. Keywords had to appear at least 30 times to be selected (Figure 10A). The most common keyword was “insulin resistance” (*N* = 2283), followed by “inflammation” (*N* = 1409) and “obesity” (*N* = 1271). Four keywords (“insulin resistance,” “inflammation,” “obesity,” and “macrophages”) appeared over 1000 times. Additionally, three keywords, “activation,” “adipose tissue,” and “expression” appeared over 500 times. We also utilized the R package “bibliometrix” to analyze popular topics from 1999 to 2023, which can display yearly keywords, research trends, and topic dynamics (Figure 10B). The research mainly focused on “obesity,” “activation,” and “inflammation” with “insulin resistance” being the most frequent term.

**Table 7:** Top 10 keywords on the research of macrophage polarization in IR.

Rank	Keyword	Occurrences
1	Insulin resistance	2283
2	Inflammation	1409
3	Obesity	1271
4	Macrophage	1027
5	Activation	907
6	Adipose‐tissue	713
7	Expression	692
8	Mice	325
9	Necrosis‐factor‐alpha	312
10	Cells	303

## 4 Discussion

### 4.1 General Information

Before 1999, there were no publications in the field of macrophage polarization in insulin resistance (IR), indicating that this area had not been explored during that time. Relevant articles began to emerge in 1999, and from 1999 to 2009, the field was still in its early stages, with an average of 29 papers published per year. From 2010 to 2017, the number of publications showed a significant upward trend, averaging 206 papers per year. This surge suggests that the field of macrophage polarization in IR entered a period of rapid growth, attracting increased attention from researchers worldwide.

An analysis of countries and institutions reveals the dominance of the United States (USA) and its institutions in this field, publishing the highest number of articles. The top three institutions are all based in the USA. The USA also engages in close collaboration with other countries, having the maximum total link strength. Consequently, China, as the second‐leading country in this field, has the opportunity to enhance the quantity and quality of documents. Additionally, the analysis suggests a relatively limited number of joint studies involving multiple countries, emphasizing the need for increased international connection and collaboration in future studies.

In terms of journals, *Journal of Clinical Investigation* is the most co‐cited, while *Diabetes* stands out as the most prolific in document publications and is the second most co‐cited journal. Considering the number of documents and co‐citations, *Diabetes* emerges as a key journal for in‐depth study in the field of macrophage polarization in IR. Furthermore, *PLOS ONE* and the *Journal of Biological Chemistry* are gaining prominence as emerging and popular journals, ranking in the top 10 for both article publications and co‐citations in the field of macrophage polarization in IR. Hence, these two journals also warrant thorough investigation.

Examining authors and co‐cited authors, Lumeng CN, emerges as a prominent figure in the field, publishing the most documents and being the most frequently co‐cited. Weisberg SP has made significant contributions, with his 2003 article in the *Journal of Clinical Investigation* being the top reference with the strongest citation bursts. Weisberg SP has also been co‐cited over 1,000 times, closely following Lumeng CN. Overall, these authors have played a foundational role in shaping the theory and practice of macrophage polarization in IR.

### 4.2 Knowledge Base

The analysis of co‐cited references, which are collectively cited by a diverse array of papers or publications, serves to uncover the foundational aspects of research in a given field [44]. In our bibliometric study, we used VOSviewer not only to analyze all co‐cited references but also to select the top 20 co‐cited references, aiming to elucidate the foundational research on macrophage polarization in IR. The research titled “Obesity is associated with macrophage accumulation in adipose tissue,” authored by Weisberg SP et al. and published in *Journal of Clinical Investigation* in December 2003 was the first to propose the impact of macrophage polarization on adipose tissue. Professor Weisberg SP contributed two articles to the top 20 co‐cited papers. Among the top 20 co‐cited papers, Professor Lumeng CN had the most co‐cited articles (*N* = 3), with one published in *Journal of Clinical Investigation* discussing a novel F4/80(+)CD11c(+) population of adipose tissue macrophages (ATMs) [27], and two published in *Diabetes* exploring the relationship between IR and ATMs [28].

In summary, the top 20 co‐cited references focus on topics such as the mechanism of the phenotypic switch of ATMs, the contribution of ATMs to IR, and MCP‐1 expression in adipose tissue, forming the fundamental basis for research in IR.

### 4.3 Hotspots and Frontiers

Analyzing the frequency of citations for relevant references in recent years allows us to discern emerging topics with significant citation bursts in a specific field. Our citation burst analysis reveals that the processes and types of macrophage polarization in IR, as well as strategies to promote or alter macrophage polarization for obesity treatment, have become prominent topics in the current discourse. These are some differences from the previous bibliometric analysis that identified tumors, infection, and obesity as the most promising hotspots, while atherosclerosis and metastasis were found to be frontiers [49].

Moreover, keywords play a crucial role in identifying ongoing research hotspots in the study of macrophage polarization in IR. As shown in Table 6, noteworthy keywords include inflammation, activation, adipose‐tissue, and necrosis factor‐alpha, excluding common terms like IR, obesity, and macrophage. It shows that macrophage polarization in IR is an evolutionary process [50] and fully demonstrates the importance of inflammation in the study of this field [51].

Through the combined analysis of references with citation bursts and keyword clustering, we gain insights into the latest research on macrophage polarization in IR, primarily focusing on two key aspects: understanding the mechanisms by which macrophage polarization leads to IR and exploring treatment strategies to control macrophage polarization for managing IR.

### 4.4 Advantages

This research has distinctive merits as it employs bibliometric methods for a systematic analysis of macrophage polarization in IR, which is more specific and focused, providing insights into disease‐specific areas and offering comprehensive guidance to researchers in this field. Furthermore, we utilized four bibliometric tools: VOSviewer, CiteSpace, The R package “bibliometrix,” and Tableau Public. The first three are widely recognized for processing bibliometric data, ensuring the objectivity and accuracy of our study, and broadening the depth and breadth of the analysis. Finally, this analysis was conducted over a longer time frame, providing insights into recent research while offering intuitive perspectives and predictions for future research hotspots. It serves as a convenient tool for scholars to identify research directions. This study can provide valuable clues for further research related to insulin resistance and immune inflammation.

### 4.5 Shortcomings

At the same time, the study has certain limitations. The first limitation is that it exclusively relies on articles and data sourced from the WoSCC, potentially overlooking relevant articles and data available in other databases. Secondly, our filtering criteria, which limit articles to English‐written reviews from 1999 to 2023, may lead to an underestimation of articles that do not meet these specific conditions, including nonreview articles, those published outside the specified timeframe, and articles not in English. This may limit the comprehensiveness of our study in this field. Lastly, compared with other reported studies, the focus of this article on specific areas may result in a lack of a macro perspective on the overall research trend of macrophage polarization.

## 5 Conclusion

In summary, the role of macrophage polarization in IR continues to receive constant attention, with numerous articles and reviews being published. In the upcoming years, a key research focus will be on regulating macrophage polarization to prevent IR and its associated symptoms and conditions. At the same time, the various types of macrophages will also garner widespread attention in IR and may become a hot spot in the future. Through this bibliometric analysis, we hope to summarize and analyze the articles published in this field thus far, to gain insight into current hot information. We aim to enhance understanding of this field, identify potential areas of interest, and discern future directions. This, in turn, can contribute to strengthening collaboration across regions and countries.

## Author Contributions

**Chuning Lin:** formal analysis; investigation; software; visualization; writing–original draft. **Yuan Chen:** Conceptualization; data curation. **Yao Ge:** conceptualization; data curation. **Huimin Niu:** investigation; validation. **Xinyi Zhang:** investigation; validation. **Feng Jiang:** funding acquisition; resources. **Chuyan Wu:** project administration; supervision; writing–review & editing.

## Conflicts of Interest

The authors declare no conflicts of interest.

## Data Availability

The original contributions presented in the study are included in the article. Further inquiries can be directed to the corresponding authors.
